# Therapeutic Effects of Electrical Stimulation: Interpretations and Predictions Based on the Visceral Theory of Sleep

**DOI:** 10.3389/fnins.2018.00065

**Published:** 2018-02-12

**Authors:** Ivan N. Pigarev, Marina L. Pigareva

**Affiliations:** ^1^Institute for Information Transmission Problems (Kharkevich Institute), Russian Academy of Sciences, Moscow, Russia; ^2^Institute of Higher Nervous Activity and Neurophysiology, Russian Academy of Sciences, Moscow, Russia

**Keywords:** electrical Stimulation, visceral theory of sleep, Sleep pressure, visceral nerves, sleepiness

Attempts to use electrical stimulation for treatment of various diseases began even before the nature of both, nervous system and electricity, were discovered (e.g., Devinsky, 1993). Later scientific interpretations of therapeutic effects of electrical stimulations have been based on the functional properties of neural circuits established for wake conditions. Here we discuss a different set of mechanisms for the stimulation therapy, which emerge from our recent investigations into the visceral theory of sleep (Pigarev, 2014).

This theory is based on the observations that, during sleep, neurons in various cortical areas, including the primary visual cortex (area V1), switched from the processing of exteroceptive information (visual, somatosensory and so on) to processing of signals coming from various visceral organs like the stomach, intestine and cardiovascular and respiratory systems (Pigarev, 1994; Pigarev et al., 2006, 2013; Pigarev and Pigareva, 2014). Taking into account these observations, we proposed that, during sleep, the cerebral cortex and other brain regions perform maintenance of body organs, including the brain itself, and generate the host responses to any pathological deviations in their states.

It is well known that sleep deprivation leads to unavoidable death of animals (i.e., Everson et al., 1989), and it is generally recognized that impairments of sleep are connected with various visceral disorders in humans (for reviews see i.e., Knutson and Van Cauter, 2008; Pigarev and Pigareva, 2012; Ali et al., 2013; Surani, 2014). On the other hand, improvement of disturbed sleep or restoration of normal sleep patterns has a therapeutic effect in the case of various diseases (Cizza et al., 2010; Chaput and Tremblay, 2012). But how can this be related to electrical stimulation? Actually, this link is rather direct, and is connected with the probable mechanism of transition from wakefulness to sleep. Details of this approach were presented in our previous study (Pigarev and Pigareva, 2013). Briefly, the transition from wakefulness to sleep and back, we consider, as the balance between two groups of needs—needs of wakefulness and needs of sleep. Needs of wakefulness are determined by the activity of an organism in the surrounding environment, and are based on information coming from extero- and proprio-receptors. Needs of sleep are determined by the states of all visceral organs of the organism, and are based on interoceptive information. But which kind of interoceptive information will increase the need for sleep and provoke sleepiness? If the purpose of sleep is to restore functionality of an organism, it would be logical to propose that needs of sleep should be related to interoceptive error signals. While all interoceptive parameters are within the genetically determined normal range, organisms may stay awake and continue to realize their needs of wakefulness. However, as soon as these parameters decline from safe values, this abnormality will be perceived as tiredness and necessity to sleep. Thus, deviation of the current visceral afferentation from the normal range should provoke sleepiness.

Based on these considerations, it is reasonable to suggest that electrical stimulation could mimic visceral sensory signals and contribute to the transitions between the awake and sleep states. In the first studies where electrical stimulations were applied, it was naturally considered that such stimulation could produce functional responses normally associated with the nervous structure being activated. However, later it became clear that electrical stimulations disrupts normal activity of neuronal circuits instead of activating them in a meaningful way. Even being applied to the peripheral nerves, skin or muscles, electrical stimulation provokes absolutely artificial combinations of fiber activation, which can hardly be imagined in any natural conditions. We should remember that in all parts of a body, thousands of terminals of interoceptive nerves are distributed, and electrical stimulation will excite them also in uncommon ways. This uncommon stimulation of the visceral afferents will be considered as error signals, and will increase the need for sleep. In particular cases sleep might be an immediate reaction, but more likely this stimulation will just increase the need for sleep, which may improve the quality of sleep during the subsequent night or nights. This improved sleep, but not the stimulation itself, may lead to more efficient body restoration, and in the case of any diseases, will facilitate treatment. Thus practically any electrical stimulation potentially may cause nonspecific general therapeutic effects.

The somnolent effect of visceral nerve stimulation was noticed long ago, even before the discovery of electricity. It was known that mechanical stimulation of a neck along the carotid artery evoked sleep—the carotid artery is sometimes even called the “sleep artery.” Now we know that this effect is related to stimulation of the vagus nerve, which passes along the carotid artery. In animal experiments it was shown that electrical stimulation of the vagus nerve did indeed provoke sleep (Juhasz et al., 1985). But in order to promote sleep it is not necessary to stimulate all of a nerve. Local stimulation of some peripheral branches might also be sufficient.

In our previous study, we investigated neuronal activity in the cat visual cortex during sleep, and applied visceral electrical stimuli in the area of the small intestine (Pigarev, 1994). We were afraid that intraperitoneal electrical stimulation could decrease the depth of sleep. However, we soon noticed that intestinal stimulation, on the contrary, shifted sleep to an even deeper level. Later we found that this somnolent effect of intestinal stimulation had already been noticed and described by Hungarian scientists (Kukorelli and Juhasz, 1976).

Sleep-promoting effects of visceral stimulation, as with any other responses of the nervous system, are habituating. The strongest effect is observed in response to first presentations. This effect will be less on the next day, and it can disappear after several days of application. An interval of non-stimulation for a couple of weeks restores the initial reaction. We have not studied these habituation effects systematically, and we mention them only because permanent changing of the stimulating parameters might be useful to avoid such habituation.

It is noteworthy that direct brain stimulation in certain cases can promote sleep and even act as anesthesia. While these effects are mostly based on the specific functions of the stimulated brain structures, some contribution is possible from the nonspecific mechanisms similar to the described above.

We found only one study where electrical stimulation of skin surface was efficiently used for improvement of sleep quality (Indursky et al., 2013). However, for this purpose the acupuncture - a mechanical counterpart of electrical stimulation is often used. In many special studies it was shown that acupuncture is effective in improvement of sleep quality and treatment of sleep disorders. See, for example, Cao et al. (2009), Bosch et al. (2014) and Fu et al. (2017). Within the frame of the visceral theory of sleep we also have proposed that acupuncture efficiency for treatment of various health problems, at least partly, could be related with improvement of sleep quality after acupuncture sessions (Pigarev, 2014).

The sleep-inducing effect could be minor compared to the other positive effects of electrical stimulation. But some impact of increased sleepiness can be expected in many cases of such stimulation. It would be very useful if investigators could pay special attention to the quality of sleep of their patients before and after stimulating procedures.

The high probability of growing sleepiness after procedures of electrical stimulation should be taken into account if patients have to use cars after these procedures, because increased risk of car accidents can be expected. On the other hand it might be reasonable to try to use electrical stimulations distributed in various locations over the body, and having variable combinations of stimulating parameters. If our theoretical considerations are correct, one may expect to get a substantial somnolent effect of this procedure, probably capable of replacing, in some cases, the use of pharmacological agents, with the added benefit of being free from the often unpleasant side effects of these drugs.

## Author contributions

All authors listed have made a substantial, direct and intellectual contribution to the work, and approved it for publication.

### Conflict of interest statement

The authors declare that the research was conducted in the absence of any commercial or financial relationships that could be construed as a potential conflict of interest.
